# Recombinant inbred lines and next-generation sequencing enable rapid identification of candidate genes involved in morphological and agronomic traits in foxtail millet

**DOI:** 10.1038/s41598-021-04012-1

**Published:** 2022-01-07

**Authors:** Kenji Fukunaga, Akira Abe, Yohei Mukainari, Kaho Komori, Keisuke Tanaka, Akari Fujihara, Hiroki Yaegashi, Michie Kobayashi, Kazue Ito, Takanori Ohsako, Makoto Kawase

**Affiliations:** 1 Faculty of Life and Environmental Sciences, Prefectural University of Hiroshima, 5562 Nanatsuka-cho, Shobara, Hiroshima 727-0023 Japan; 2 Iwate Biotechnology Research Center, 22-174-4 Narita, Kitakami, Iwate 024-0003 Japan; 3 NODAI Genome Research Center, Tokyo University of Agriculture, 1-1-1 Sakuragaoka, Setagaya-ku, Tokyo, 156-8502 Japan; 4 Graduate School of Life and Environmental Sciences, Kyoto Prefectural University, 74 Kitainayazuma, Seika-cho, Kyoto, 619-0244 Japan; 5 Tokyo University of Agriculture, 1737 Funako, Atsugi, Kanagawa 243-0034 Japan; 6 Present Address: Institute of Agrobiological Sciences, Institute of Agrobiological Sciences, National Agriculture and Food Research Organization (NARO), 3-1-1 Kannondai, Tsukuba, Ibaraki 305-0856 Japan

**Keywords:** Evolution, Genetics, Plant sciences

## Abstract

We constructed recombinant inbred lines (RILs) between a Japanese and a Taiwanese landrace of foxtail millet and employed next-generation sequencing, such as flexible ddRAD-seq and Nanopore sequencing to identify the candidate genes involved in the crop evolution of foxtail millet. We successfully constructed a linkage map using flexible ddRAD-seq with parents and RILs and detected major QTLs for each of three traits: leaf sheath colors, spikelet-tipped bristles (stb), and days to heading (DTH). (1) For leaf sheath colors, we identified the *C* gene on chromosome IV. (2) We identified a homeobox (*HOX14*) gene for stb on chromosome II, which shows homology with *HvVrs1* in barley. (3) Finally, we identified a QTL with a large effect on DTH on chromosome II. A parent of the RILs from Taiwan and Yugu1 had a Harbinger-like TE in intron 3 of this gene. We also investigated the geographical distribution of the TE insertion type of this gene and found that the insertion type is distributed in the northern part of East Asia and intensively in South and Southeast Asia, suggesting that loss/reduction of function of this gene plays an important role in spreading into the northern part of East Asia and subtropical and tropical zones.

## Introduction

Foxtail millet [*Setaria italica* (L.) P. Beauv.] is one of the oldest cereals in the Old World. It is characterized by diploidy with small chromosome numbers (2n = 2x = 18), small genome size (approximately 500 Mb), an inbreeding habit, and a relatively short growth habit; therefore, it has become an ideal model plant for genetic studies on panicoid grass species, including switchgrass and Napier grass, which are considered as biofuel sources, and other cultivated millet species such as pearl millet^1^. This millet adapts to various environmental conditions, and its agronomic traits show large variation as a result of adaptation to local environments ranging from temperate to tropical climates, high- and low-altitude conditions, and cultivation under various cultural conditions. Several landrace groups are genetically differentiated and distributed in different geographical areas^2^. The foxtail millet genome has been sequenced^3,4^ and recently, the genome of its presumed wild ancestor, *S. viridis*, was also determined^5^. Owing to its high variations in several agronomic traits, this millet will also be a good material for studying crop evolution in the context of adaptation to variable environmental conditions and human selection.

Recently, next-generation sequencing (NGS) technology has become a powerful tool for genetic mapping and population genetics. Several methods for genetic mapping using NGS have also been developed, including genotyping by sequencing (GBS) ^6,7^, RAD-seq^8,9^, ddRAD-seq^10,11^, QTL-seq^12^, and GRAS-Di^13,14^.

Several studies on gene and QTL mapping^15–23^ and genome-wide association studies (GWAS)^24–26^ in foxtail millet have been conducted using NGS technology, and several genes have been mapped and identified successfully. However, most studies on QTL mapping have been carried out using hybrid-derived populations from crosses within Chinese cultivars, Japanese cultivars, and between *Setaria viridis* and a Chinese cultivar. No population between distantly related landraces collected from different geographical conditions has been used for mapping.

**Figure 1:**
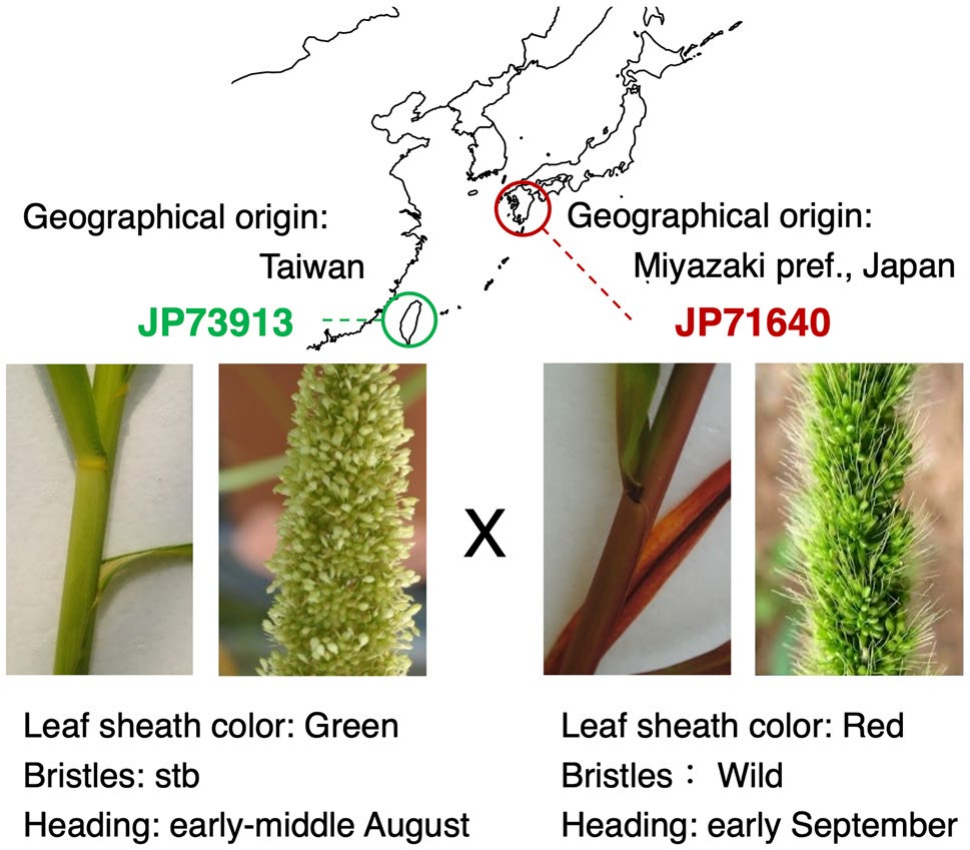
Characteristics of parental landraces. Maternal parent: JP73913 from Taiwan with green leaf sheath and spikelet-tipped bristles (stb) and heading early-middle August. Pollen donor: JP71640 from Miyazaki Prefecture, Japan, with red leaf sheath and wild type of bristles and heading early September. The map was created using the R package “maps” ver. 3.3.0 (https://CRAN.R-project.org/package=maps).

Consequently, we constructed an RIL population derived from a cross between a Japanese landrace (JP71640) and a Taiwanese landrace (JP73913) adapting to different latitudes and climates and mapped three traits: leaf sheath color, spikelet-tipped bristles (stb), and days to heading (DTH) (Fig. 1). JP 71640 and JP 73913 were chosen as parents because they are genetically distinct from each other in terms of morphological and physiological characteristics, rDNA genotypes, RFLP variations of genomic DNA, and intervarietal hybrid sterility^2^. Herein, we successfully identified major QTLs and identified candidate genes for the *C* gene controlling leaf sheath color, a homeobox (*HOX14*) gene for stb, and *PRR37* for DTH. We also found allelic variation in *the C* gene and the homeobox gene in Yugu1 and naturally occurring mutants of *S. viridis*, respectively. The geographical distribution of the TE-insertion type of *S. italica PRR37* (*SiPRR37*) will be discussed in terms of its adaptive significance.

## Results

### Segregation of traits

**Figure 2:**
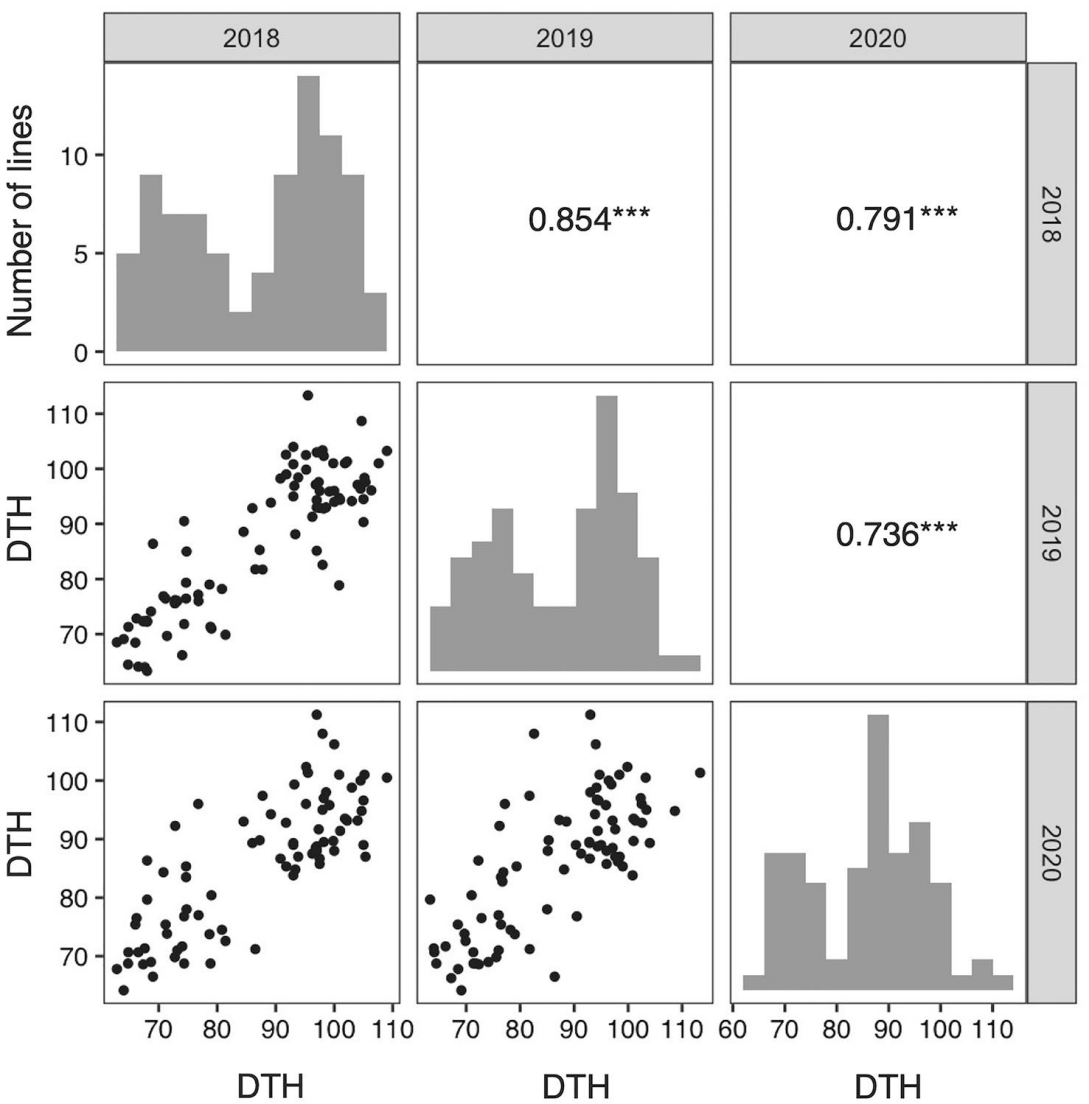
Histograms showing variation of DTH in foxtail millet RILs in 2018, 2019, and 2020 and correlation between different years. The diagonal from the upper left to the lower right : Histograms showing variation DTH in foxtail millet. Upper right: Correlation coefficients between different years. ***P < 0.001. Lower left: Scatter diagrams of DTH of RILs between different years.

Stb and the green leaf sheath are recessive traits^27^, as we previously reported in the F_2_ population. Two types of leaf sheath colors, red and green, were observed in 49 and 41 lines of the RILs, respectively. This result fits the 1:1 ratio using the Chi-square test (*P* > 0.05). Common wild-type bristles and mutant stb (Supplementary Fig. S1) are observed in lines 43 and 47, respectively. This result also fits the 1:1 by Chi-square test (*P* > 0.05). Days to heading (DTH) showed a rather broad but bimodal distribution in all cultivations in 2018, 2019, and 2020, and a high positive correlation in DTH between different years was observed (Fig. 2).

### Construction of high-density linkage maps

**Figure 3:**
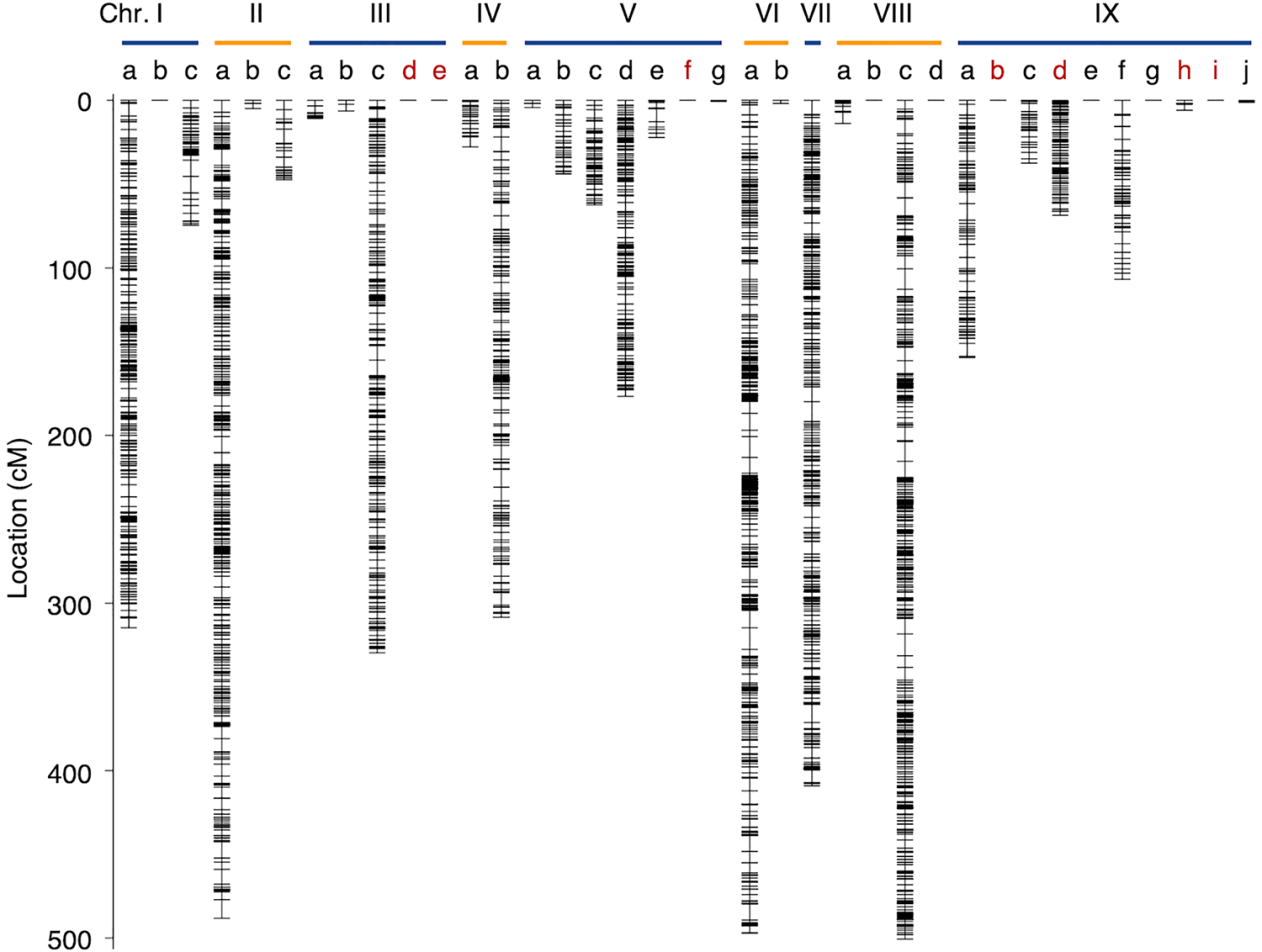
A high-density linkage map of the RILs of foxtail millet based on the flexible ddRAD-seq. Chromosome numbers correspond to *S. italica* reference genome, GCF_000263155.2, and the alphabets mean linkage group. The linkage group with red alphabets has been constructed using markers that the segregation distortion does not fit the expected ratio (1:1).

The genomes of all RILs for the F_10_ generation were subjected to flexible ddRAD-seq to detect genome-wide SNPs and Presence/Absence (PAs) markers, which were then used to construct a high-density linkage map. Following the quality-control steps, a total of 6,818 SNPs and 3,430 PAs were detected using ddRAD-seq. By adding 153 SSR/INDEL markers to these, 37 linkage groups corresponding to chromosomes I-IV^3^ were constructed (Fig. 3, Supplementary Table S1). As shown in previous research^20–22^, the S-shape correlation of markers between physical distances and genetic distances was observed (Supplementary Fig. S2).

### QTL mapping and identification of candidate gene

#### Leaf-sheath color

**Figure 4:**
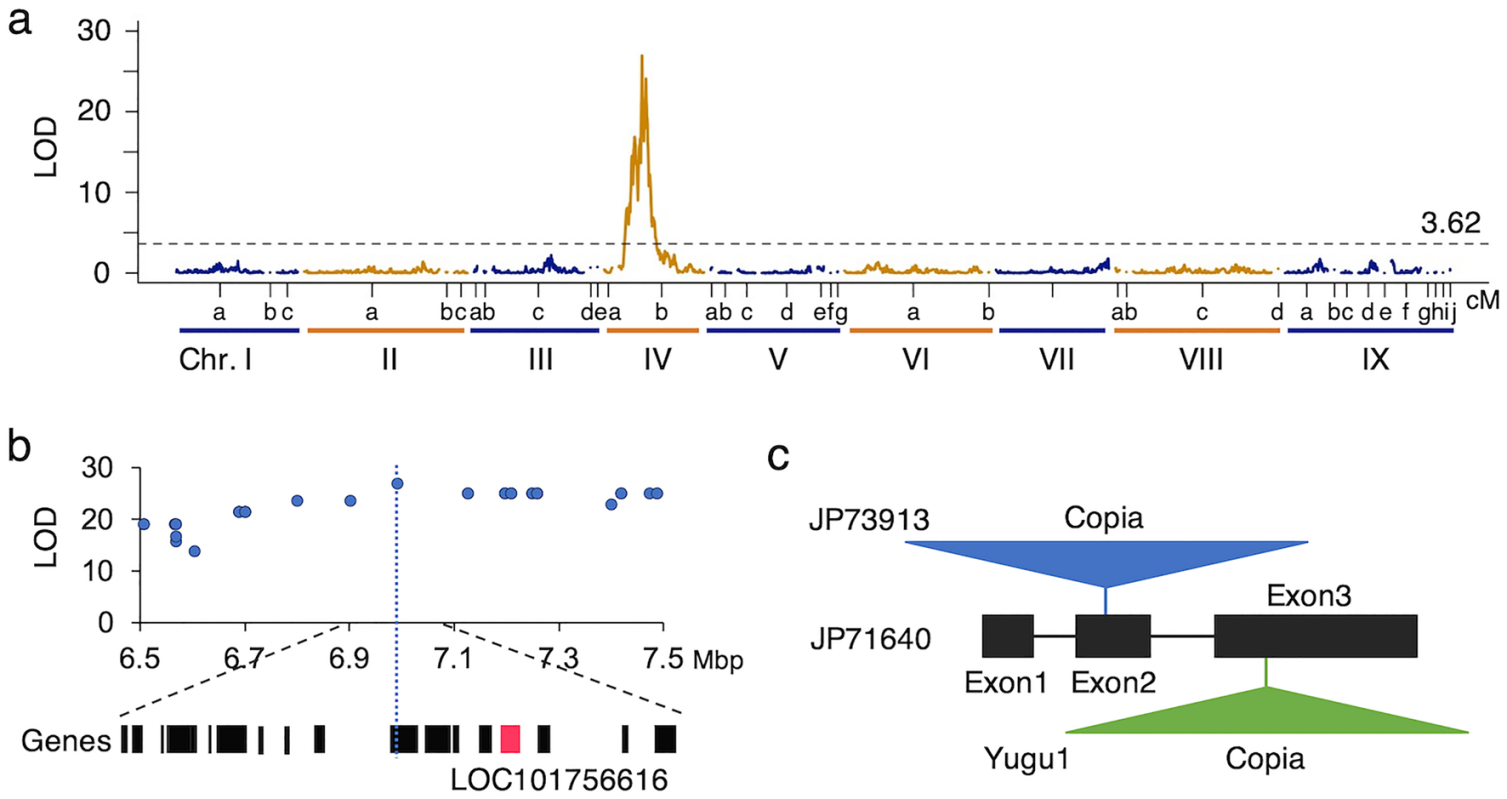
Analysis of leaf sheath color of the foxtail millet. (**a**) Distribution of LOD scores for the leaf sheath colors in RILs. (**b**) Enlarged view of the physical map around the marker which showed the highest LOD score. The annotated genes on the *S. italica* reference genome are shown at the bottom part, and the red one is *C* gene which is the candidate gene for leaf sheath color. (**c**) Structure of *C* gene for leaf sheath color. Transposable element (TE) insertions each for JP 73913 with green sheath and Yugu1 with green leaf sheath.

The QTL analysis detected a genomic region strongly associated with leaf sheath color in linkage group IVb, which corresponds to chromosome IV, as expected in a previous study^27^ (Fig. 4a, Supplementary Table S2). A candidate gene, *C*, which encodes the anthocyanin regulatory C1 protein (LOC101756616), was closely located with a marker showing the highest LOD score (Fig. 4b), as Jia et al.^24^ suggested. We compared the genome sequence between JP71640 with a red leaf sheath and JP73913 with a green leaf sheath. JP71640 has a complete *C* gene sequence, but JP73913 has a large insertion (ca. 6.5 kb) in exon 2 (Fig. 4c). It is inferred that this gene loses its function in the green leaf sheath. Interestingly, another accession, Otsuchi10 with red leaf sheath, had no insertion in this gene, but Yugu1 and Nisatai-zairai, with a green leaf sheath, had a large insertion (ca. 5.1 kb) in exon 3 (Fig. 4c). Homology search using Censor (http://www.girinst.org/censor/index.php) showed that a large insertion found in exon 2 is a *Copia*-type retrotransposon (*Copia* 6 Sit), and the other found in exon 3 is another *Copia*-type retrotransposon (*Copia* 20 Sit). It is strongly suggested that these transposable element (TE) insertions are causal mutations in the green leaf sheath.

#### Stb

**Figure 5:**
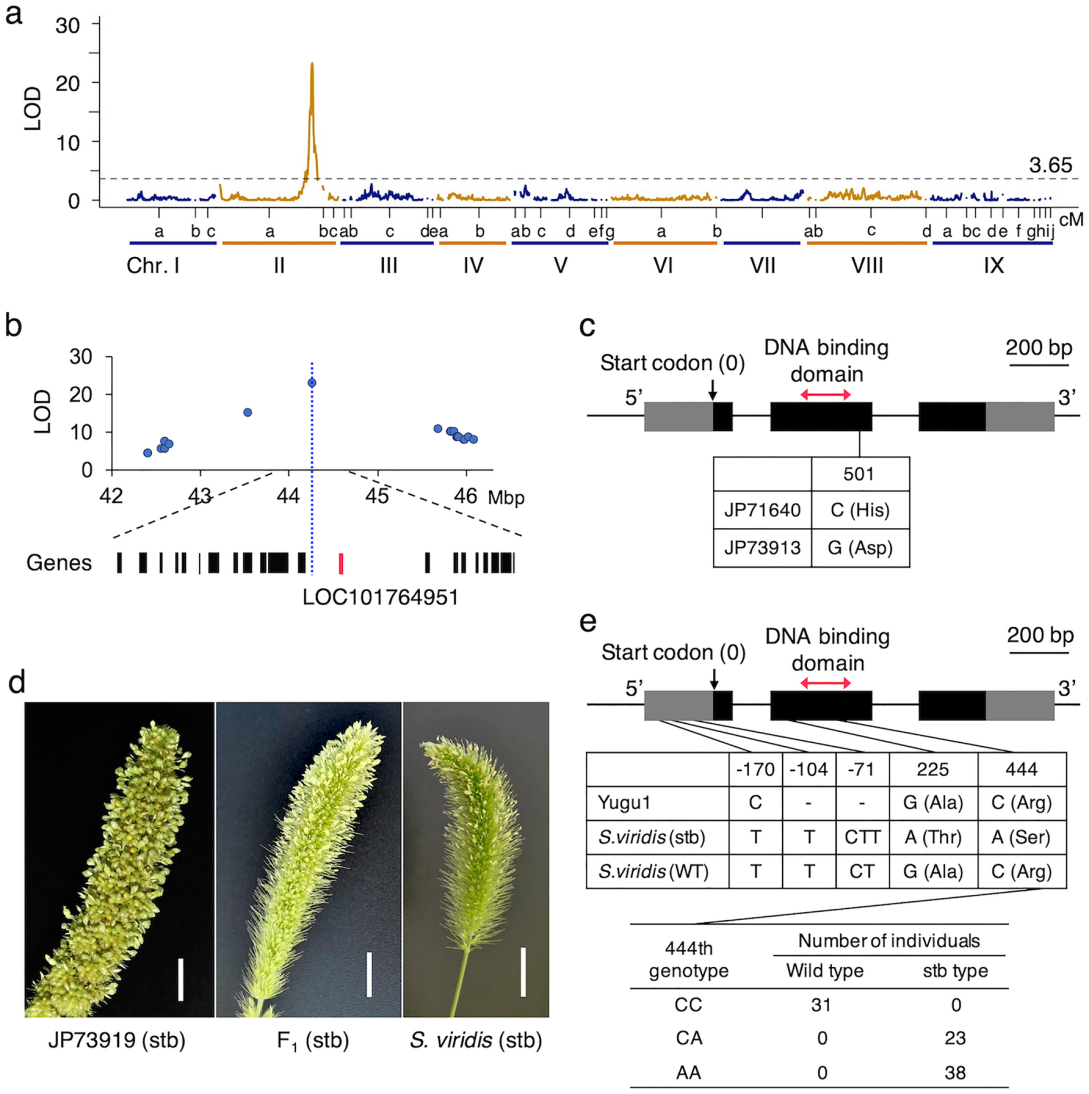
Analysis of spikelet tipped bristles (stb) in foxtail millet. (**a**) Distribution of LOD scores for stb in RILs. (**b**) Enlarged view of the physical map around the marker which showed the highest LOD score. The annotated genes on the *S. italica* reference genome are shown at the bottom part, and the red one is *HOX14* which is the candidate gene for stb. (**c**) Structure of *HOX14* gene, a candidate gene for stb phenotype. A non-synonymous SNP presents in the exon 2. (**d**) Allelism test using cross between JP73913 and ‘Kyoto stb-1’ that is stb mutant of *S. viridis*. These two accessions and F_1_ hybrid between them showed stb phenotype. White bar indicates 10 mm. (**e**) Structure of *HOX14* gene. Two SNPs specific to Kyoto stb-1 (*S. viridis*) are present in the exon 2 (middle table). The reference genome sequence of *S. viridis*, A10.1^5^, was used as the wild-type (WT) sequence. The 444th SNP genotype in F_3_ individuals derived from a cross between Yugu1 and ‘Kyoto stb-1’ correspond to stb phenotype (bottom table).

A genomic region associated with the stb phenotype was detected on chromosome II, as in our previous study^27^ (Fig. 5a, Supplementary Table S2). A homeobox-leucine zipper protein *HOX14* (LOC101764951), which is a homolog of *HvVrs1*, and encodes a transcription factor responsible for the evolution of two-rowed barley to six-rowed barley^28^, was found 14 kb downstream of the SNP with the highest LOD score (Fig. 5b). Comparison of the genomic sequences of *HOX14* between the parental accessions (JP73913 and JP71640) revealed an SNP at the 501st position of *HOX14,* causing an amino acid substitution from histidine to aspartic acid (Fig. 5c).

To confirm that the causative gene was *HOX14*, we performed an allelism test using a naturally occurring stb mutant of *S. viridis* (Supplementary Fig. S3), which we discovered in Japan, and designated "Kyoto stb-1." It seems that this *S. viridis* mutant occurred independently of JP73913. We crossed it with JP73913 to successfully obtain F_1_ hybrids. The F_1_ individuals also showed an stb phenotype, suggesting that this *S. viridis* mutant is allelic to JP73913 in the stb causative gene (Fig. 5d). We sequenced the *HOX14* gene of Yugu1 and Kyoto stb-1 and found two SNPs in the coding region, which altered the amino acid sequences of the protein, although these two SNPs were not present in wild-type *S. viridis*, A10.1^5^ (Fig. 5e, DDBJ:LC642768). Furthermore, we investigated the co-segregation of the stb phenotype and genotype, which was genotyped using the KASP genotyping assay, at 444th position in the DNA binding domain of HOX14 in 92 F_3_ individuals derived from a cross between Yugu1 and ‘Kyoto stb-1’ (Supplementary Fig. S4). As shown in Fig. 5e, 38 individuals had the apparent stb phenotype with the AA genotype of the 444th SNP, and thirty-one individuals with the CC genotype of the SNP had the wild type of bristles. Twenty-three individuals had the wild type of bristles with the CA genotype (of the 23 individuals, 12 had only a few stbs on the tip of the panicle, probably because of heterozygosity (Supplementary Fig. S5), suggesting that this gene controls the trait.

#### DTH

**Figure 6:**
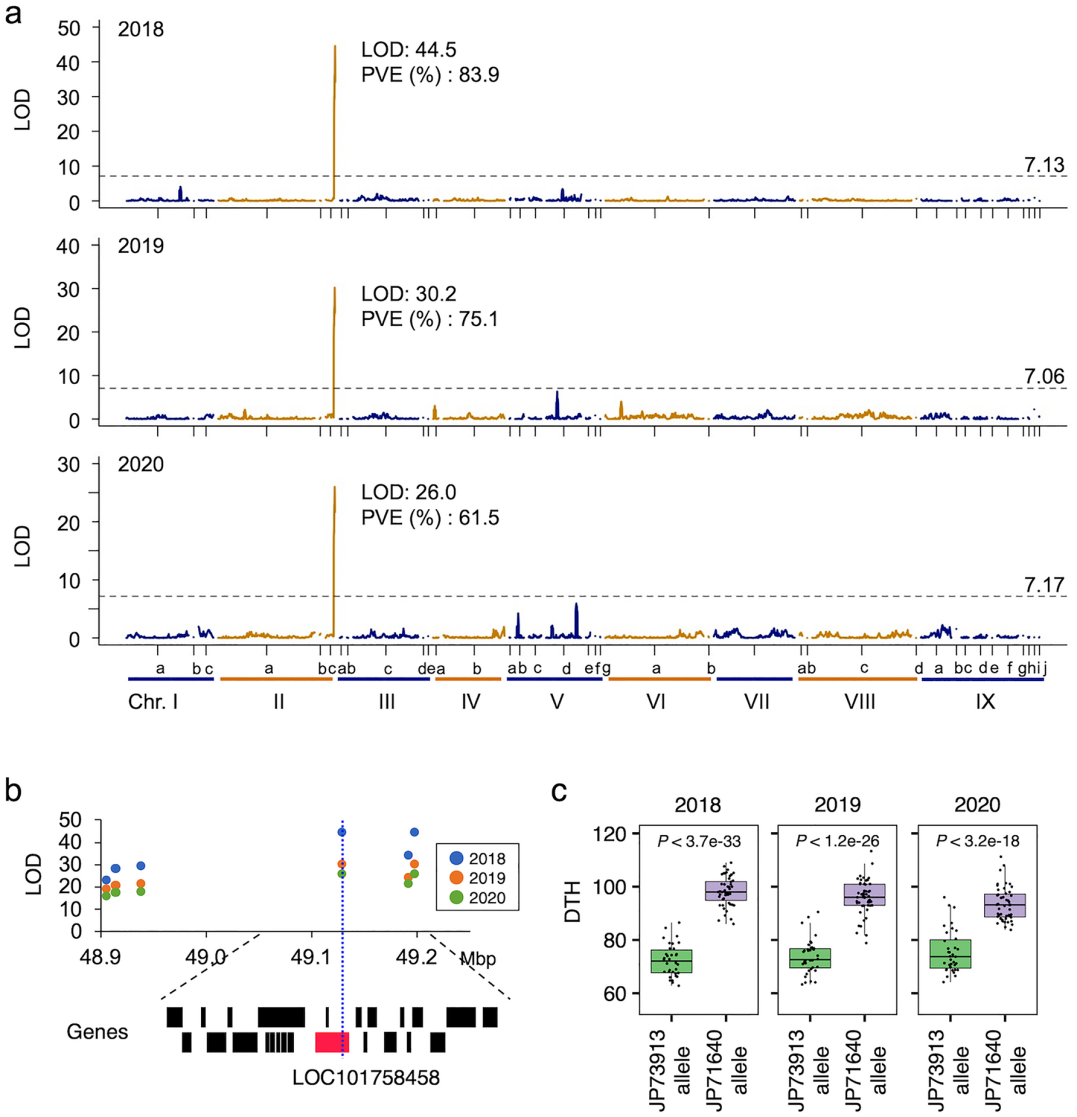
Analysis of days to heading (DTH) in foxtail millet. (**a**) Distribution of LOD scores for RILs in three years. PVE indicates the phenotypic variation explained by QTL. (**b**) Enlarged view of the physical map around the QTL. The annotated genes on the *S. italica* reference genome are shown at the bottom part, and the red one is *SiPRR37* which is the candidate gene for DTH. (**c**) Box and dot plots of DTH between the different genotypes (JP73913 homozygous allele and JP71640 homozygous allele) at the marker on *SiPRR37*. *P*-values obtained by the student's two-sample t-test function in R are shown in each panel to assess statistical significance between the two genotypes of the RILs.

Composite interval mapping (CIM) based on the data obtained from individual cultivations for the three years shown in Fig. 2 indicates that there is a QTL with a very high LOD score (Fig. 6a). Consequently, a QTL with a very high LOD score and a high percentage of phenotypic variation explained by QTL (PVE) values on chromosome II was commonly found for three years (Fig. 6a, Supplementary Table S2). One of the SNPs, NC_028451.1_49128686_A_C_0_2 marker, with a higher LOD score was located on a gene (LOC101758458) that showed homology to *PRR37*, which is involved in DTH in several grass species such as rice^29^, sorghum^30^, wheat^31^, and barley^32^. It has also been reported to be important in latitudinal adaptation (Fig. 6b). To clarify the association between DTH and the NC_028451.1_49128686_A_C_0_2 marker, the distribution of DTH values for genotypes of RILs at this marker is shown in Fig. 6c. The DTH comparisons between the JP73913 and JP71640 genotypes demonstrated an association between DTH and genotype every year. The JP73913 genotype for this marker accelerated heading, while the JP71640 genotype delayed it, with a difference of about 20 days between them (Fig. 6c).

#### Geographical distribution of the TE-insertion-type *SiPRR37* gene

**Figure 7:**
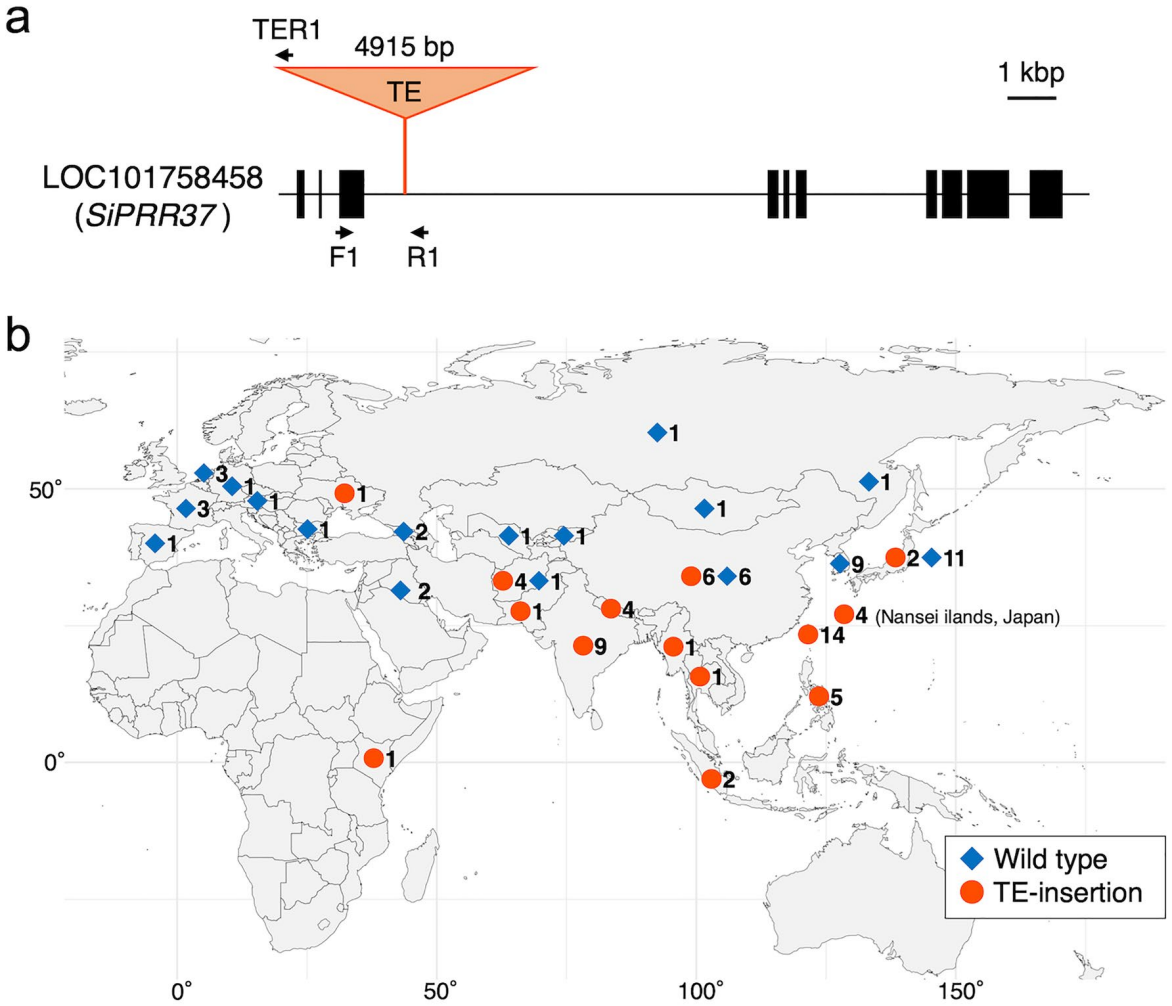
The variant of *SiPRR37* with TE-insertion and its geographical distribution. (**a**) Structure of the *SiPRR37* gene and insertion of transposable element (TE). Primers used for the analysis of TE-insertion were shown as arrows. (**b**) Geographical distribution of TE-insertion type and non-insertion type (Wild type) of *SiPRR37* gene of foxtail millet. The number next to the symbol indicates the number of accessions checked in this study. The map was created using the R package “maps” ver. 3.3.0 (https://CRAN.R-project.org/package=maps). The X-axis and the Y-axis indicate longitude and latitude, respectively.

Comparison of the genomic sequences of *SiPRR37* between the parental accessions (JP73913 and JP71640) revealed a TE-insertion in the *SiPRR37* intron of JP73913 (Fig. 7a). Yugu1, a variety of reference sequences for foxtail millet, also had a TE insertion in the same way. Recently, Li et al.^26^ also found that this gene, *SiPRR37*, is key to adaptation in northeast China by GWAS, and they also reported TE-insertion in this gene. The TE found in this study and Li et al*.*^26^ are the same sequence and inserted in the same position in the gene, whereas they referred to the TE found in their study as *Tc1–Marine*. We also investigated the geographical distribution of the TE-insertion type of this gene in 99 accessions, as shown in Fig. 7b. Among the 99 accessions investigated, 55 were of the TE-insertion type, whereas 44 were not (Supplementary Table S3). The TE-insertion type is mostly distributed in tropical and subtropical regions and parts of East Asia. Contrarily, the non-insertion type is widely distributed in temperate zones. This insertion type was distributed in six of 12 accessions from China, and intensively in the Nansei-Islands of Japan, Taiwan, and Southeast Asia, including the Philippines, Indonesia, Thailand, and Myanmar; South Asia such as Nepal, India, and Pakistan; four accessions of Afghanistan; one accession of Ukraine; and one from Kenya. Two Japanese accessions from the northeastern part of Japan (Iwate Prefecture) also had this TE insertion. Specifically, the non-insertion-type *SiPRR37* gene is mainly distributed in Japan, Korea, China, Central Asia, and Europe. Further analysis of this gene to heading will be required to understand adaptation of this millet to the latidude.

We found no TE-insertion of the *SiPRR37* gene for all six accessions of *S. viridis* analyzed in this study.

## Discussion

We constructed RILs between JP 71640 and JP 73913, a Japanese and Taiwanese landrace of foxtail millets, which are phylogenetically and genetically distinct^2^. Various combinations of the characters between the parents were observed in cultivation experiments (data not shown), and the RILs constructed should be novel useful tools for crop evolutionary studies and breeding new varieties.

The flexible ddRAD-seq method employed in this study was highly effective for constructing a fine linkage map. We efficiently obtained more than 10,000 markers in this linkage map using flexible ddRAD-seq, whereas we obtained only 138 DNA markers, including SSR and transposable display (TD) markers^27^ in the F_2_ population between the same cross combination. This RIL population genotyped by flexible ddRAD-seq rapidly identified the candidate genes involved in this study’s morphological and agronomical traits. This population with a high density map will be useful for further mapping studies of other traits which were not investigated in the present study. In the present study, we used the MinION sequencer (Oxford Nanopore Technologies), in addition to the HiSeq X platform (Illumina). The de novo assembly using a combination of these platforms was also very practical for identifying the insertion of transposable elements into the gene.

We identified the candidate gene *C* gene, an MYB transcriptional factor for leaf sheath color, as suggested by Jia et al.^24^. Homologs of this gene have been reported to be involved in the pigmentation of many cereal plants such as rice, wheat, barley, and maize^33–35^. JP73913, a maternal parent of the RILs, has a *Copia* TE inserted in exon 2 of the gene, apparently causing loss of function of the gene. For Yugu1, a reference genome sequence of foxtail millet^3^ with a green leaf sheath, another copy TE was inserted in exon 3 of the gene, causing loss of gene function. These mutations caused by different TE insertions onto the same gene, which changed the red leaf sheath to green, should have occurred independently. Multiple origins of loss-of-function genes have been reported in waxy^36,37^ and polyphenol oxidase^38^ in foxtail millet. The green leaf sheath is also a good example of the multiple origins of the loss-of-function phenotype in foxtail millet. We are now investigating the origin of the green leaf sheath using more landraces from various Eurasian regions. The details will be published elsewhere because the origin of the green sheath seems more complicated.

Developing bristles in the genus *Setaria*, including foxtail millet, is important in the evolution of the bristle clade in the family Poaceae^27^. *Stb1* mapped on chromosome II, as reported in our previous study^27^ and is located close to a homeobox (*HOX14*) gene, LOC101764951, a homolog of *HvVrs1*, the gene responsible for the evolution of two-rowed barley to six-rowed barley^28^. As *HvVrs1* is a transcription factor involved in the development (suppression) of lateral spikelets in two-rowed barley, it is more likely that this homeobox gene suppresses the development of spikelets from the bristles and is functionally similar to the *HvVrs1* gene.

We found that JP73913 with stb had a non-synonymous SNP in this gene but not in the homeobox domain. It is also possible that some SNPs and indels in the 5’ region upstream of the coding sequences may alter the gene expression pattern. We confirmed that a naturally occurring mutant ‘Kyoto stb-1’ gene is allelic to that of JP73913. We sequenced the coding sequence of the homeobox gene ‘Kyoto stb-1*’* and found two SNPs in the coding sequences (Fig. 5e). We confirmed stb and SNP in F_3_ individuals derived from Yugu1 and ‘Kyoto stb-1’ (Fig. 5e). Knockout of this gene by genome editing will be useful to confirm that this gene is responsible for stb.

For DTH, a QTL with a large effect was consistently found in the same region on chromosome II in all three-year trials. The *SiPRR37* gene is located at the SNP with the highest LOD score (LOD value: 2018; 44.5, 2019; 30.2, 2020; 26.0). Interestingly, JP73913 has a Harbinger TE insertion in intron 3, whereas JP71640 does not. Yugu 1 also had a TE insertion in this intron. Takei and Sakamoto^39,40^ reported that variation in the heading date of foxtail millet is determined by the length of basic vegetative growth and sensitivity to daylength. Mauro-Herrera et al.^15^, and Yoshitsu et al*.*^23^ investigated QTLs on DTH in foxtail millet and found some QTLs such as *qDTH2* and *qDTH7* but did not suggest the *SiPRR37* gene in their mapping populations. Recently, Li et al.^26^ reported that *SiPRR37* plays an important role in the adaptation of Chinese cultivars to high latitudinal regions in China. In this study, we also found a large LOD peak of QTL on chromosome II in our population and found that *SiPRR37* is a candidate gene for the difference in DTH between the parents. A TE was inserted in this gene of JP73913, which is the same as that reported by Li et al*.*^26^. The analyses of 99 accessions covering the whole traditional distribution area of foxtail millet landraces revealed that TE-insertion type is mainly distributed in parts of Japan and China and subtropical and tropical regions of Eurasia, such as Taiwan, Southeast Asia, and South Asia. Ranges of DTH of landraces showing non-TE-insertion type (34–116 days) and that of landraces with TE-insertion types of *SiPRR37* gene (42–169 days ) overlapped in our data obtained in cultivation experiment in Shobara, Hiroshima, in 2011^42^ (Supplementary Table S3) but TE-insertion type is mainly distributed in the middle latitudal region. In the middle latitudinal region, photosensitivity to daylength is important in the adaptation of landraces, whereas high-latitude and low-latitude region sensitivity to daylength are not always necessary^39,40^. TE-insertion into the *SiPRR37* gene leading to loss or reduction of function of the gene is essential for the spread of foxtail millet in low-latitude areas such as the tropical and subtropical regions, as found in this study and high latitudinal areas in China, as reported by Li et al.^26^. *SiPRR37* plays important role in adapting to latitudinal areas in other cereals, such as rice^29^ and sorghum^30^. This work strongly suggests that insertion of the TE in *SiPRR37* is key to elucidating adaptation to not only high latitude but also low latitudinal areas in Asia. Interestingly, six accessions of green foxtail, *S. viridis*, did not have this insertion in the gene, although the number of accessions is still limited. Insertion of the TE in the *SiPRR37* gene might have occurred after domestication. Further analysis of more accessions of green foxtail and foxtail millet will reveal the spread of TE-insertion type in Eurasia.

The naturally occurring splicing variant *Heading date1* (*HD1*) gene, an ortholog of *CONSTANS* of Arabidopsis, was reported in foxtail millet^41,42^. Although JP71640 is wild type and JP73913 is a splicing variation according to Fukunaga et al*.*^42^, no QTL was detected in this analysis. This suggests that the splicing variant of *HD1* may not contribute to the DTH between the two parents. Further analyses of genes involved in DTH and interaction between the genes are required to understand adaptation of foxtail millet to various ecological condition of Eurasia in detail.

TEs have been reported to play important roles in foxtail millet domestication and diversification, for example, MITE insertion upstream of the *Sh1* gene^19^ and *Copia* insertion in the *SvLes1* gene^5^ involved in seed shattering, insertion of 11 different TEs in *waxy* genes involved in the control of amylose content in endosperm^36,37^, Harbinger transposon, and LINE insertion in the *Si7PPO* gene involved in phenol color reaction^38^. In addition to these genes, DTH was drastically changed by TE insertion in *SiPRR37*, and leaf sheath color was also changed by multiple independent insertions of *Copia* TEs in the *C* gene. It seems that several TEs were involved in the domestication and diversification of foxtail millet. Further analysis of genome-wide TE insertions will enable an in-depth understanding of crop evolution in foxtail millet. Analysis of active TEs will also be useful in reverse genetics in foxtail millet and *S. viridis* as used for transposon-tagging in maize and rice^43,44^.

## Methods

### Ethics statement

The authors declare that all methods were performed in accordance with the relevant guidelines and regulations. The test materials were provided by NARO Genebank, Japan, Iwate Agricultural Research Center (IARC), Japan, USDA and Dr. Katrien Devos, University of Georgia, Athens (UGA). We obtained permission from the Genabank, IARC, USDA and UGA to use these accessions.

### Plant materials and mapping population

We used a Taiwanese landrace (NARO Genebank JP 73913) that has spikelet-tipped bristles (stb) phenotype^45^, green leaf sheath, and heading in early-middle August was used as the maternal parent (Fig. 1). This landrace is the only accession with spikelet-tipped bristles among foxtail millet collections in the NARO Genebank, although this trait was reported in a collection of foxtail millet in India^46^, and is a naturally occurring mutant in a Japanese *Setaria viridis* population, as shown later. A Japanese landrace (JP71640) with wild-type bristles, red leaf sheath, and heading in early September (Fig. 1) was used as a pollen donor. The F_2_ population was obtained as described by Sato et al.^27^, and recombinant inbred lines (RILs) were constructed by a single seed descent (SSD) method^47,48^. In this study, we used 90 individuals of RIL (F_9_/F_10_) populations for linkage map construction and QTL mapping.

For whole-genome sequencing, we also used Otsuchi10 and Nisatai-zairai in addition to JP73913 and JP71640. These accessions are breeding materials for foxtail millet in Iwate Prefecture in the northern part of Japan.

A naturally occurring stb mutant of the wild ancestor, *S. viridis*, was discovered by TO in Kumihama, Kyotango City, Kyoto Prefecture and designated as ‘Kyoto stb-1’ mutant. The ‘Kyoto stb-1’ was used for an allelism test of the gene in F_1_ hybrid with JP73913. We crossed the ‘Kyoto stb-1’ with Yugu1 (standard accession of foxtail millet for reference sequence^3^) after checking the complementation of the gene with JP73913, and developed F_2_ and F_3_ populations by self-pollination.

We used 99 accessions of foxtail millet and 6 accessions of *S.viridis* for genotyping of *SiPRR37* gene (Supplementary Table S3). All the foxtail millet accessions except two Japanese accessions (Otsuchi10 and Nisatai-zairai) and Yugu1 were provided by NARO Genebank, Japan. Otsuchi10 and Nisatai-zairai were provided by Iwate Agricultural Research Center (IARC) and Yugu1 was provided by Dr. K. Devos, University of Georgia, Athens, USA. All the six accessions of *S.viridis* were provided by USDA.

### Cultivation of RILs and the investigation of traits

In 2018, 2019, and 2020, we cultivated parents and RILs in the Experimental Farm, Field Science Center, Prefectural University of Hiroshima, Shobara, Hiroshima, Japan (34.83°N, 132.98°E). In 2018 and 2020, six individuals for each line were cultivated in pots, and in 2019, 13–14 individuals were cultivated in a greenhouse (15 cm between individuals in a row and 30 cm between rows) under natural day length. Seeds were sown on June 6, 2018, May 23, 2019, and June 4, 2020, respectively. The heading date was checked as described by Fukunaga et al.^42^. Stb and leaf sheath color were checked according to Sato et al.^27^.

### DNA extraction

For genotyping, Sanger sequencing, and Illumina sequencing, we used the DNeasy Plant Mini Kit (QIAGEN) for DNA extraction according to the supplier’s recommendations. To extract high molecular weight DNA from leaf tissue for nanopore sequencing, we used NucleoBond HMW DNA (Takara) with some modifications. After DNA extraction, low molecular weight DNA was eliminated using the Short Read Eliminator Kit XL (Circulomics). DNA of F_3_ individuals derived from a cross between Yugu1 and ‘Kyoto stb-1’ was extracted from dried leaves using a buffer containing 0.2 M Tris-HCl, 0.25 M NaCl, 25 mM EDTA, and 0.5% SDS followed by 2-propanol precipitation^38^.

### Flexible ddRAD-seq and RIL genotyping

We used flexible ddRAD-seq to construct a linkage map^49^. We designed all enzymatic reactions to be completed sequentially without DNA purification in each step to make the procedures simple^49^. Additionally, we designed 92 sets of indexed and forked sequencing adaptors compatible with Illumina platform sequencers. The RAD-seq reads were qualified using Trimmomatic (ver. 0.39)^50^. Only ‘paired’ output reads were retained, and the read sizes used in the subsequent analysis were approximately 742 Mb in JP73913, 758 Mb in JP71640, and 441–830 Mb in RILs. These reads were mapped onto the reference genome of *Setaria italica* (Accession: PRJNA32913, Assembly: GCF_000263155.2, Cultivar: Yugu1)^3^ using the ‘bwa mem’ command in BWA (ver. 0.7.12)^51^. SNP-based genotypes for JP73913 and JP71640, and each RIL was obtained as a variant call format (VCF) file. The VCF file was generated from BAM files of JP73913, JP71640, and each RIL using SAMtools (ver 1.5)^52^, and the VCF variants were called and filtered using BCFtools (ver 1.5)^52^. Presence/Absence-type genotypes are also called by the following method: First, the VCF file was generated from BAM files of JP73913 and JP71640 and selected the region where either JP73913 or JP71640 had sufficient read depth (≥ 8) and that the other parental line had no read depth in that region. Next, BEDtools (ver 2.26)^53^ converted continuous positions in the VCF file to a feature, and only sufficiently wide features (width ≥ 50 bp) were retained as the BED file. For these regions in the BED file, the RIL genotypes were classified into three categories (depth ≥ 2, depth = 0, others) and three genotypes (“existence,” “absence,” “NA”). In this study, we rejected the features with a high percentage of ‘NA’ (0.3 or higher). Additional details are provided in the Supplementary Methods section.

### Linkage map construction

For the segregation distortion analysis in each marker using the genotypes obtained in the previous section, the Chi-square test was used to calculate the deviation from the expected ratio (1:1) for each marker. The significance level was adjusted using the Bonferroni correction to account for multiple comparisons. Markers that fit the 1:1 expected ratio and markers that did not fit the expected ratio were used to construct the linkage group separately. The genetic linkage maps were constructed using MSTmap^54^ with the following parameters: “population_type RIL10; distance_function kosambi; cut_off_p_value 0.0000000000002; no_map_dist 15.0; no_map_size 0; missing_threshold 0.25; estimation_before_clustering no; detect_bad_data no; objective_function ML.” The linkage groups were visualized by the R/qtl package^55^. The number of linkage groups was the same as that used in the *S. italica* reference genome^3^.

### QTL analysis

QTL analysis for days to heading (DTH) was performed using the composite interval mapping (CIM) function of the R/qtl package^55^ with the Haley–Knott regression method^56^. For the qualitative traits, leaf sheath color, and spikelet-tipped bristles (stb), QTL analysis was performed using the scanone function with option “model = binary.” The LOD significance threshold for detecting QTLs was calculated by performing 1,000 iterations using the R/qtl permutation test. The position and effect of significant QTLs were assessed for percentage phenotypic variation explained (PVE%) by fitting a model containing all QTLs identified for a given trait in R/qtl.

### de novo assembly of* S. italica* accessions

Sequencing of the RIL parents (JP71640 and JP73913) and two other landraces, Otsuchi10 and Nisatai-zairai from Iwate Prefecture, Japan, were carried out using Illumina HiSeq X for short reads and Nanopore MinION (R9.4 flow cell) for long reads. Base calling of nanopore long reads was performed on FAST5 files using Guppy (ONT, UK). The nanopore long reads, which were converted to FASTQ format, were subsequently assembled using NECAT^57^. To further improve the accuracy of the assembly, Racon^58^ was used twice, and Medaka (ONT) was subsequently used to correct misassembly. Following this, one round of consensus correction was performed using BWA^51^ and HyPo^59^ with Illumina short reads. Finally, we obtained the target gene sequences of the four landraces from each de novo assembly using BLAST^60^. Target gene sequences of four landraces and Yugu1 were retrieved from the whole genome sequences and aligned with the A10.1 *S. viridis* reference sequence using BioEdit^61^ and Clustal W^62^.

### Allelism test and segregation of *stb1* in *Setaria viridis*

For *stb1*, we tested allelism with a naturally occurring stb mutant of *Setaria viridis*, ‘Kyoto stb-1.’ ‘Kyoto stb-1’ was crossed with JP73913 showing stb trait in 2012. ‘Kyoto stb-1’ was used as a pollen donor and JP73913 was used as a female parent. F_1_ was cultivated in pots in 2013, and the bristles were investigated.

*HOX14* gene of ‘Kyoto stb-1’ was amplified by a primer combination hbxF1and hbxR3 and sequenced using hbxF1, hbxR3, and internal primers (Supplementary Table S4). ‘Kyoto stb-1’ was also crossed as a pollen donor with Yugu1, a maternal parent and F_1_ seeds were obtained. F_2_ and F_3_ populations were also developed in a greenhouse at the Prefectural University of Hiroshima. We confirmed that this trait was controlled by a single gene in the F_2_ population. An F_3_ population was developed and segregation of bristles and an SNP at position + 444 of *HOX14* gene (‘C’ for Yugu 1 and ‘A’ for Kyoto stb-1) was investigated. Genotyping of 92 individuals of the F_3_ population and parents was carried out using the Kompetitive Allele Specific PCR (KASP) genotyping assay (LGC Genomics). Two labeled forward primers, SihbxF-C and SihbxF-A and a reverse primer, SihbxR (Supplementary Table S4) was used for KASP genotyping assay A SNP ‘A’ was labeled with HEX whereas ‘C’ was labeled with FAM, and genotyping was carried out at LGC Genomics, UK.

### Geographical distribution of the TE insertion-type *SiPRR37* gene

To investigate the TE-insertion type of *SiPRR37* in 99 foxtail millet landraces covering a broad traditional cultivation area in Eurasia, including two parents of RILs, Yugu1, Otsuchi10, and Nisatai-zairai and six green foxtail accessions from various parts of the world (two each from Russia and one each from Kazakhstan and Chile) (Supplementary Table S3), we designed primer combinations as shown in Fig. 7a. For the TE insertion type, a ca. 300-bp band was amplified by a primer combination PRR37F1 and PRR37TER1 and a ca. 600-bp band was amplified by a primer combination PRR37F1 and PRR37R1 (Fig. 7a, Supplementary Table S4, Supplementary Fig. S6). PCR conditions were as follows:5 min at 94 °C; 35 cycles of 1 min at 94 °C, 1 min at 60 °C, and 1 min at 72 °C; and 5 min at 72 °C. Toyobo Quick Taq was used for the amplification. PCR products were run on a 1.2 % agarose gel and visualized under UV light with EtBr staining.

## Supplementary Information

Supplementary Information 1.

Supplementary Information 2.

## Data Availability

All data generated or analyzed during this study are included in the Supplementary Materials files and in the DDBJ Sequence Read Archive as shown in Supplementary Table S5.

## References

[1] Doust AN, Kellogg EA, Devos KM, Bennetzen JL (2009). Foxtail millet: A sequence-driven grass model system. Plant Physiol..

[2] Fukunaga K, Doust A, Xiao D (2017). Genetic differentiation and crop evolution of foxtail millet. Genetics and Genomics of Setaria.

[3] Bennetzen JL (2012). Reference genome sequence of the model plant Setaria. Nat. Biotechnol..

[4] Zhang G (2012). Genome sequence of foxtail millet (*Setaria italica*) provides insights into grass evolution and biofuel potential. Nat. Biotechnol..

[5] Mamidi S (2020). A genome resource for green millet *Setaria viridis* enables discovery of agronomically valuable loci. Nat. Biotechnol..

[6] Elshire RJ (2011). A robust, simple Genotyping-by-Sequencing (GBS) approach for high diversity species. PLoS ONE.

[7] Narum SR, Buerkle CA, Davey JW, Miller MR, Hohenlohe PA (2013). Genotyping-by-sequencing in ecological and conservation genomics. Mol. Ecol..

[8] Baird NA (2008). Rapid SNP discovery and genetic mapping using sequenced RAD markers. PLoS ONE.

[9] Andrews K (2016). Harnessing the power of RADseq for ecological and evolutionary genomics. Nat. Rev. Genet..

[10] Peterson BK, Weber JN, Kay EH, Fisher HS, Hoekstra HE (2012). Double digest RAD seq: An inexpensive method for de novo SNP discovery and genotyping in model and non-model species. PLoS ONE.

[11] Yang GQ (2016). Development of a universal and simplified ddRAD library preparation approach for SNP discovery and genotyping in angiosperm plants. Plant Methods.

[12] Takagi H (2013). QTL-seq: rapid mapping of quantitative trait loci in rice by whole genome resequencing of DNA from two bulked populations. Plant J..

[13] Hosoya S (2019). Random PCR-based genotyping by sequencing technology GRAS-Di (genotyping by random amplicon sequencing, direct) reveals genetic structure of mangrove fishes. Mol. Ecol. Resour..

[14] Miki Y (2020). GRAS-Di system facilitates high-density genetic map construction and QTL identification in recombinant inbred lines of the wheat progenitor *Aegilops tauschii*. Sci. Rep..

[15] Mauro-Herrera M (2013). Genetic control and comparative genomic analysis of flowering time in Setaria (Poaceae). G3.

[16] Mauro-Herrera M, Doust AN (2016). Development and genetic control of plant architecture and biomass in the panicoid grass, Setaria. PLoS ONE.

[17] Ni X (2017). Updated foxtail millet genome assembly and gene mapping of nine key agronomic traits by resequencing a RIL population. Gigascience.

[18] Zhang K (2017). Identification of QTLs for 14 agronomically important traits in *Setaria italica* based on SNPs generated from high-throughput sequencing. G3.

[19] Odonkor S (2018). QTL mapping combined with comparative analyses identified candidate genesfor reduced shattering in *Setaria italica*. Front. Plant Sci..

[20] Wang J (2017). A high-density genetic map and QTL analysis of agronomic traits in foxtail millet [*Setaria italica* (L.) P. Beauv.] using RAD-seq. PLoS ONE.

[21] Wang J (2017). Mapping of Sihc1, which controls hull color, using a high-density genetic map based on restriction site-associated DNA sequencing in foxtail millet [*Setaria italica* (L.) P. Beauv.]. Mol. Breed..

[22] Wang Z (2019). QTL mapping for 11 agronomic traits based on a genome-wide Bin-map in a large F2 population of foxtail millet (*Setaria italica* (L.) P. Beauv.). Mol. Breed..

[23] Yoshitsu Y (2017). QTL-seq analysis identifies two genomic regions determining the heading date of foxtail millet, *Setaria italica* (L.) P. Beauv. Breed. Sci..

[24] Jia G (2013). A haplotype map of genomic variations and genome-wide association studies of agronomic traits in foxtail millet (*Setaria italica*). Nat. Genet..

[25] Jaiswal V (2019). Genome-wide association study of major agronomic traits in foxtail millet (*Setaria italica* L.) using ddRAD sequencing. Sci. Rep..

[26] Li C (2021). High-depth resequencing of 312 accessions reveals the local adaptation of foxtail millet. Theor. Appl. Genet..

[27] Sato K, Mukainari Y, Naito K, Fukunaga K (2013). Construction of a foxtail millet linkage map and mapping of *spikelet-tipped bristles 1*(*stb1*) by using transposon display markers and simple sequence repeat markers with genome sequence information. Mol. Breed..

[28] Komatsuda T (2007). Six-rowed barley originated from a mutation in a homeodomain-leucine zipper I-class homeobox gene. Proc. Nat. Acad. Sci. USA.

[29] Koo BH (2013). Natural variation in *OsPRR37* regulates heading date and contributes to rice cultivation at a wide range of latitudes. Mol. Plant.

[30] Murphy RL (2011). Coincident light and clock regulation of *pseudoresponse regulator protein 37* (*PRR37*) controls photoperiodic flowering in sorghum. Proc. Nat Acad. Sci. USA.

[31] Beales J, Turner A, Griffiths S, Snape JW, Laurie DA (2007). A Pseudo-Response Regulator is misexpressed in the photoperiod insensitive Ppd-D1a mutant of wheat (*Triticum aestivum* L.). Theor. Appl. Genet..

[32] Turner A, Beales J, Faure S, Dunford RP, Laurie DA (2005). The pseudo-response regulator *Ppd-H1* provides adaptation to photoperiod in barley. Science.

[33] Saitoh K, Onishi K, Mikami I, Thidar K, Sano Y (2004). Allelic diversification at the *C* (*OsC1*) locus of wild and cultivated rice: nucleotide changes associated with phenotypes. Genetics.

[34] Himi E, Taketa S (2015). Isolation of candidate genes for the barley *Ant1* and wheat *Rc* genes controlling anthocyanin pigmentation in different vegetative tissues. Mol. Genet. Genom..

[35] Cone KC, Burr FA, Burr B (1986). Molecular analysis of the maize anthocyanin regulatory locus C1. Proc. Natl. Acad. Sci. USA.

[36] Fukunaga K, Kawase M, Kato K (2002). Structural variation in the Waxy gene and differentiation in foxtail millet [*Setaria italica* (L.) P. Beauv.]: Implications for multiple origins of the waxy phenotype. Mol. Genet. Genom..

[37] Kawase M, Fukunaga K, Kato K (2005). Diverse origins of waxy foxtail millet crops in East and Southeast Asia mediated by multiple transposable element insertions. Mol. Genet. Genom..

[38] Inoue T (2015). Multiple origins of the phenol reaction negative phenotype in foxtail millet, *Setaria italica* (L.) P. Beauv., were caused by independent loss-of-function mutations of the polyphenol oxidase (Si7PPO) gene during domestication. Mol. Genet. Genom..

[39] Takei E, Sakamoto S (1987). Geographical variation of heading response to daylength in foxtail millet (*Setaria italica* P. Beauv.). Jpn. J. Breed..

[40] Takei E, Sakamoto S (1989). Further analysis of geographical variation of heading response todaylength in foxtail millet (*Setaria italica* P. Beauv.). Jpn. J. Breed..

[41] Liu H (2015). Parallel domestication of the *Heading Date 1* gene in cereals. Mol. Biol. Evol..

[42] Fukunaga K (2015). A nucleotide substitution at the 5′splice site of intron 1 of rice *HEADING DATE 1* (*HD1*) gene homolog in foxtail millet, broadly found in landraces from Europe and Asia. Crop. J..

[43] Brutnell TP (2002). Transposon tagging in maize. Funct. Integr. Genom..

[44] Hirochika H (2010). Insertional mutagenesis with *Tos17* for functional analysis of rice genes. Breed. Sci..

[45] Sakamoto S (1979). Characteristics and ethnobotanical comparison of fox-tail millet (*Setaria italica* P. Beauv.) samples from southern Formosa and the Batan Islands. Bull. Nat. Mus. Ethnol..

[46] Ayyangar GNR, Narayanan TR, Rao TN (1933). The inheritance of characters in Setaria italica (Beauv.), the Italian millet, part IV Spikelet-tipped bristles. Ind. J. Agric. Sci..

[47] Goulden, C. H. Problems in plant selection in *Proceedings of the Seventh International Genetics* Congress. 132–133 (Cambridge University Press 1939).

[48] Brim CA (1966). A modified pedigree method of selection in soybeans. Crop Sci..

[49] Ando T (2018). Repeated inversions within a pannier intron drive diversification of intraspecific colour patterns of ladybird beetles. Nat. Commun..

[50] Bolger AM, Lohse M, Usadel B (2014). Trimmomatic: A flexible trimmer for Illumina Sequence Data. Bioinformatics.

[51] Li, H. Aligning sequence reads, clone sequences and assembly contigs with BWA-MEM. arXiv:1303.3997v2 [q-bio.GN] (2013).

[52] Danecek P (2021). Twelve years of SAMtools and BCFtools. GigaScience.

[53] Quinlan AR, Hall IM (2010). BEDTools: A flexible suite of utilities for comparing genomic features. Bioinformatics.

[54] Wu Y, Bhat PR, Close TJ, Lonardi S (2008). Efficient and accurate construction of genetic linkage maps from the minimum spanning tree of a graph. PLoS Genet.

[55] Broman KW, Wu H, Sen Ś, Churchill GA (2003). R/qtl: QTL mapping in experimental crosses. Bioinformatics.

[56] Haley C, Knott S (1992). A simple regression method for mapping quantitative trait loci in line crosses using flanking markers. Heredity.

[57] Chen, Y. *et al.* Fast and accurate assembly of Nanopore reads via progressive error correction and adaptive read selection. *bioRxiv*10.1101/2020.02.01.930107 (2020).

[58] Vaser R, Sović I, Nagarajan N, Šikić M (2017). Fast and accurate de novo genome assembly from long uncorrected reads. Genome Res..

[59] Kundu, R. Casey, J. & Sung. W. HyPo: Super fast & accurate polisher for long read genome assemblies. *bioRxiv*10.1101/2019.12.19.882506 (2019).

[60] Altschul SF, Gish W, Miller W, Myers EW, Lipman DJ (1990). Basic local alignment search tool. J. Mol. Biol..

[61] Hall TA (1999). BioEdit: A user-friendly biological sequence alignment editor and analysis program for Windows 95/98/NT. Nucleic Acids Symp. Ser..

[62] Larkin MA (2007). Clustal W and Clustal X version 2.0. Bioinformatics.

