# Supplementary material for: Recombinant inbred lines and next-generation sequencing enable rapid identification of candidate genes involved in morphological and agronomic traits in foxtail millet
Source: Sci Rep. 2022 Jan 7;12:218. doi: 10.1038/s41598-021-04012-1 (PMC8742101; doi:10.1038/s41598-021-04012-1)
Supplement: Supplementary file 2 — Supplementary Information 2. [file 41598_2021_4012_MOESM2_ESM.pptx]

## Slide 1
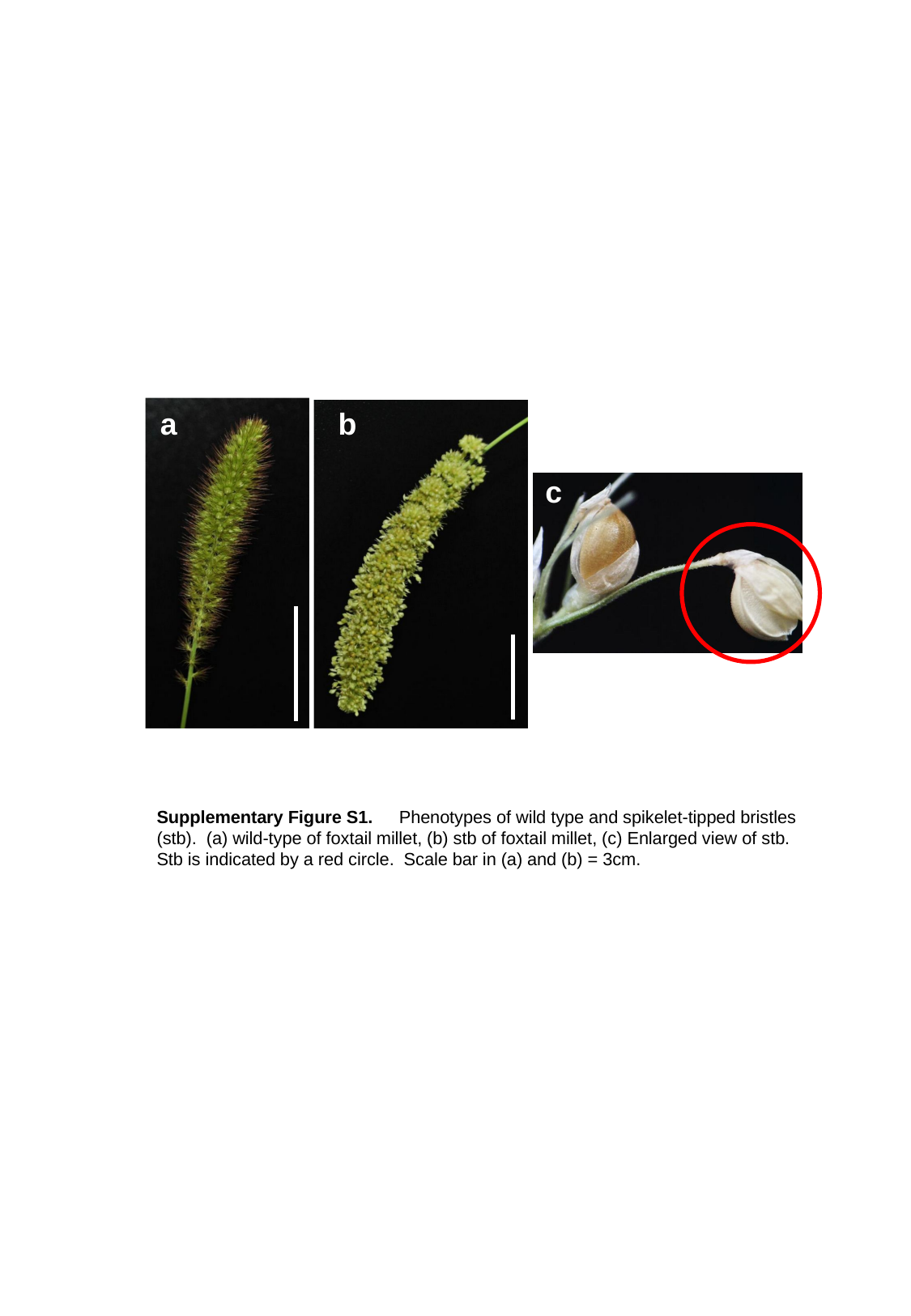

a
b
c
Supplementary Figure S1.　Phenotypes of wild type and spikelet-tipped bristles (stb). (a) wild-type of foxtail millet, (b) stb of foxtail millet, (c) Enlarged view of stb. Stb is indicated by a red circle. Scale bar in (a) and (b) = 3cm.

## Slide 2
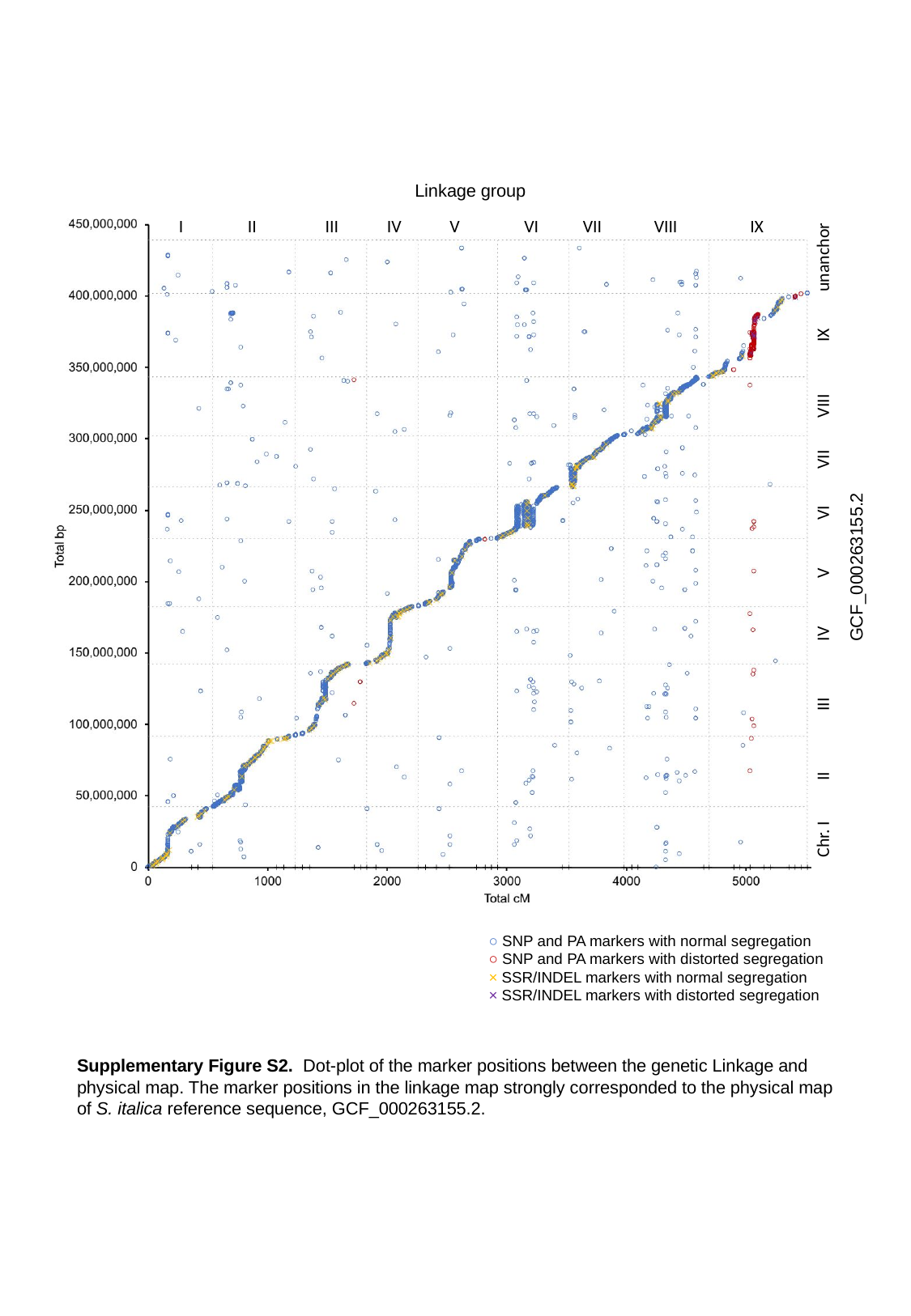

Linkage group
I II III IV V VI VII VIII IX
Chr. I II III IV V VI VII VIII IX unanchor
GCF_000263155.2
○ SNP and PA markers with normal segregation
○ SNP and PA markers with distorted segregation
× SSR/INDEL markers with normal segregation
× SSR/INDEL markers with distorted segregation
Supplementary Figure S2. Dot-plot of the marker positions between the genetic Linkage and physical map. The marker positions in the linkage map strongly corresponded to the physical map of S. italica reference sequence, GCF_000263155.2.

## Slide 3
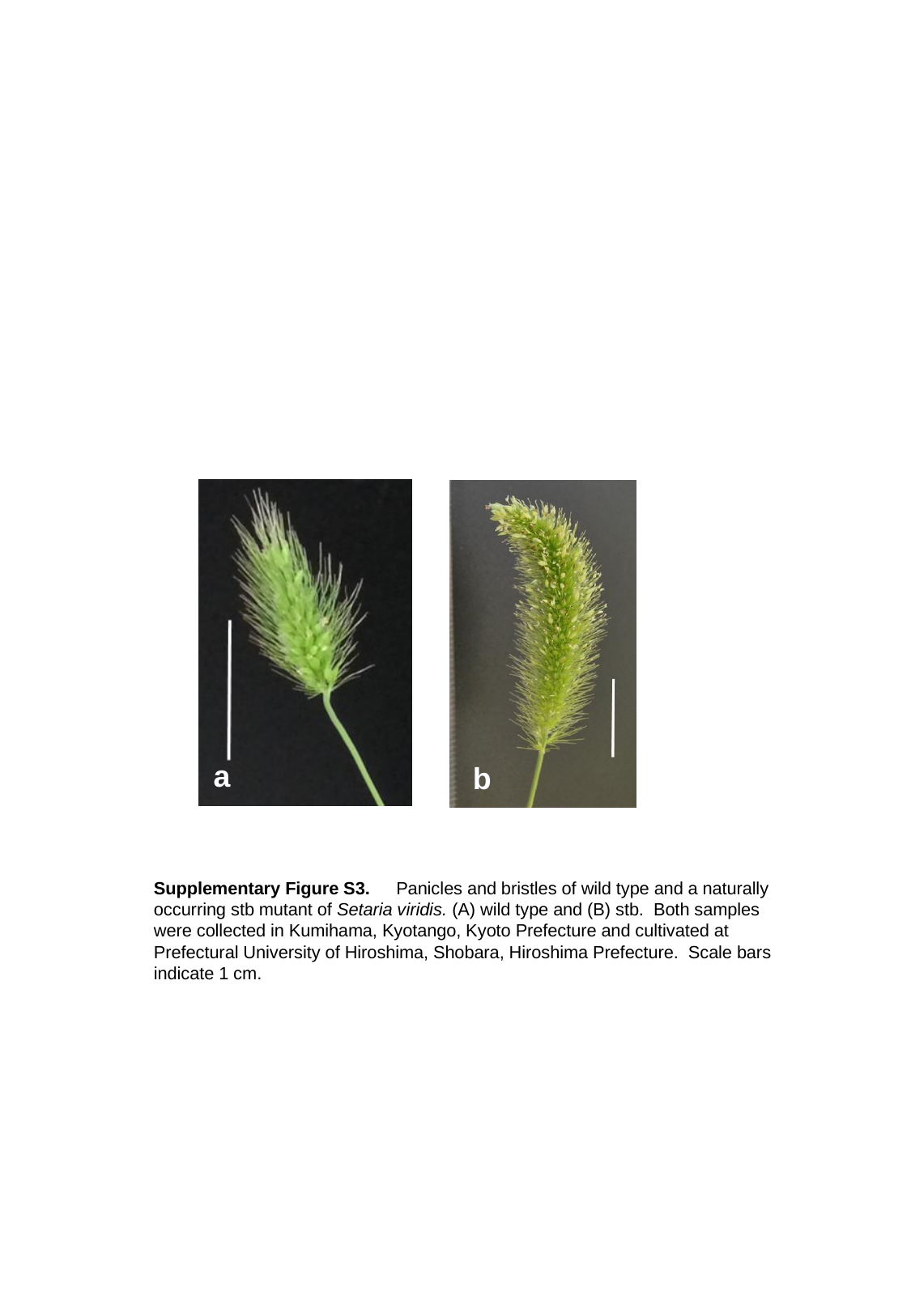

a
b
Supplementary Figure S3.　Panicles and bristles of wild type and a naturally occurring stb mutant of Setaria viridis. (A) wild type and (B) stb. Both samples were collected in Kumihama, Kyotango, Kyoto Prefecture and cultivated at Prefectural University of Hiroshima, Shobara, Hiroshima Prefecture. Scale bars indicate 1 cm.

## Slide 4
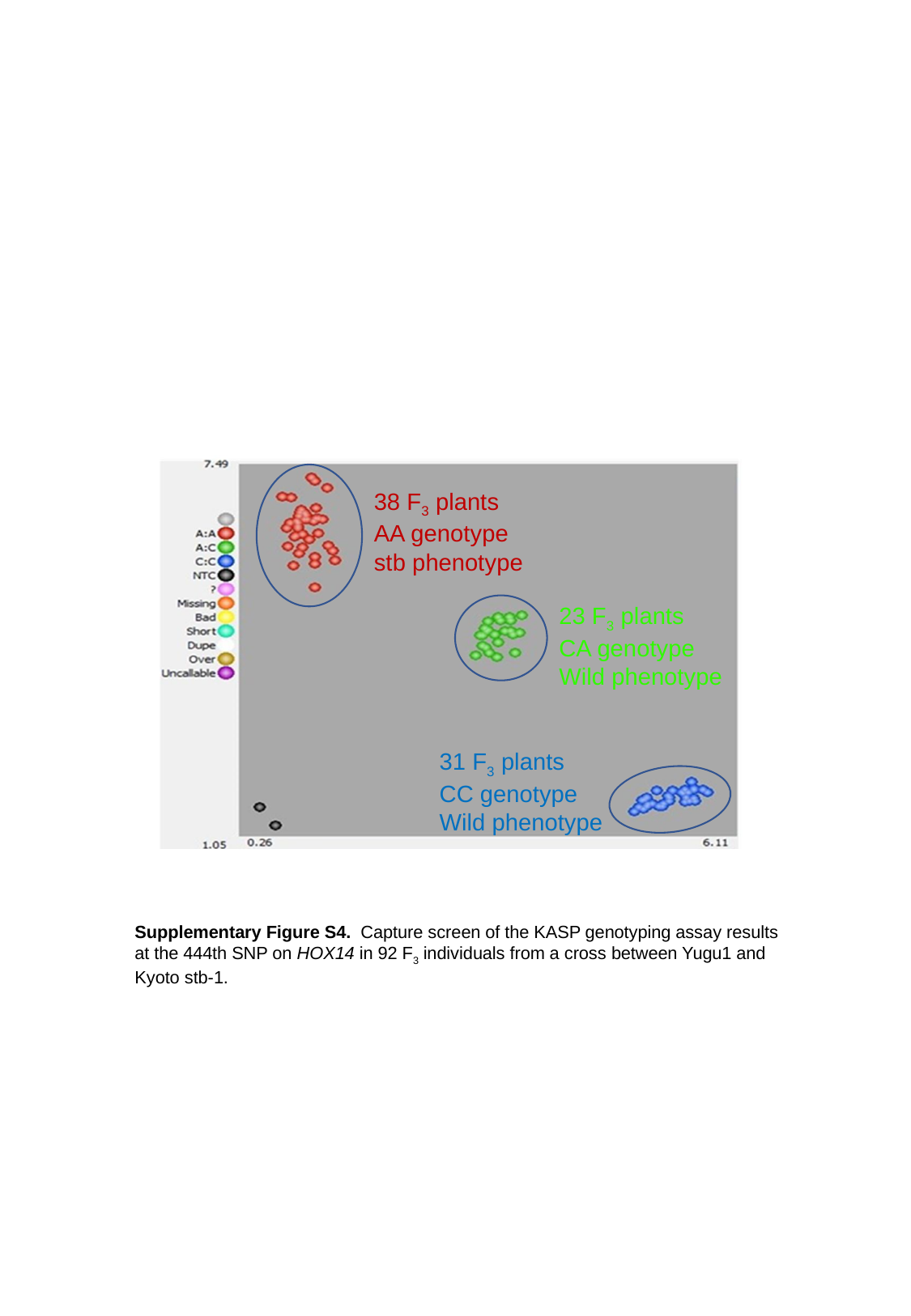

38 F3 plants
AA genotype
stb phenotype
23 F3 plants
CA genotype
Wild phenotype
31 F3 plants
CC genotype
Wild phenotype
Supplementary Figure S4. Capture screen of the KASP genotyping assay results at the 444th SNP on HOX14 in 92 F3 individuals from a cross between Yugu1 and Kyoto stb-1.

## Slide 5
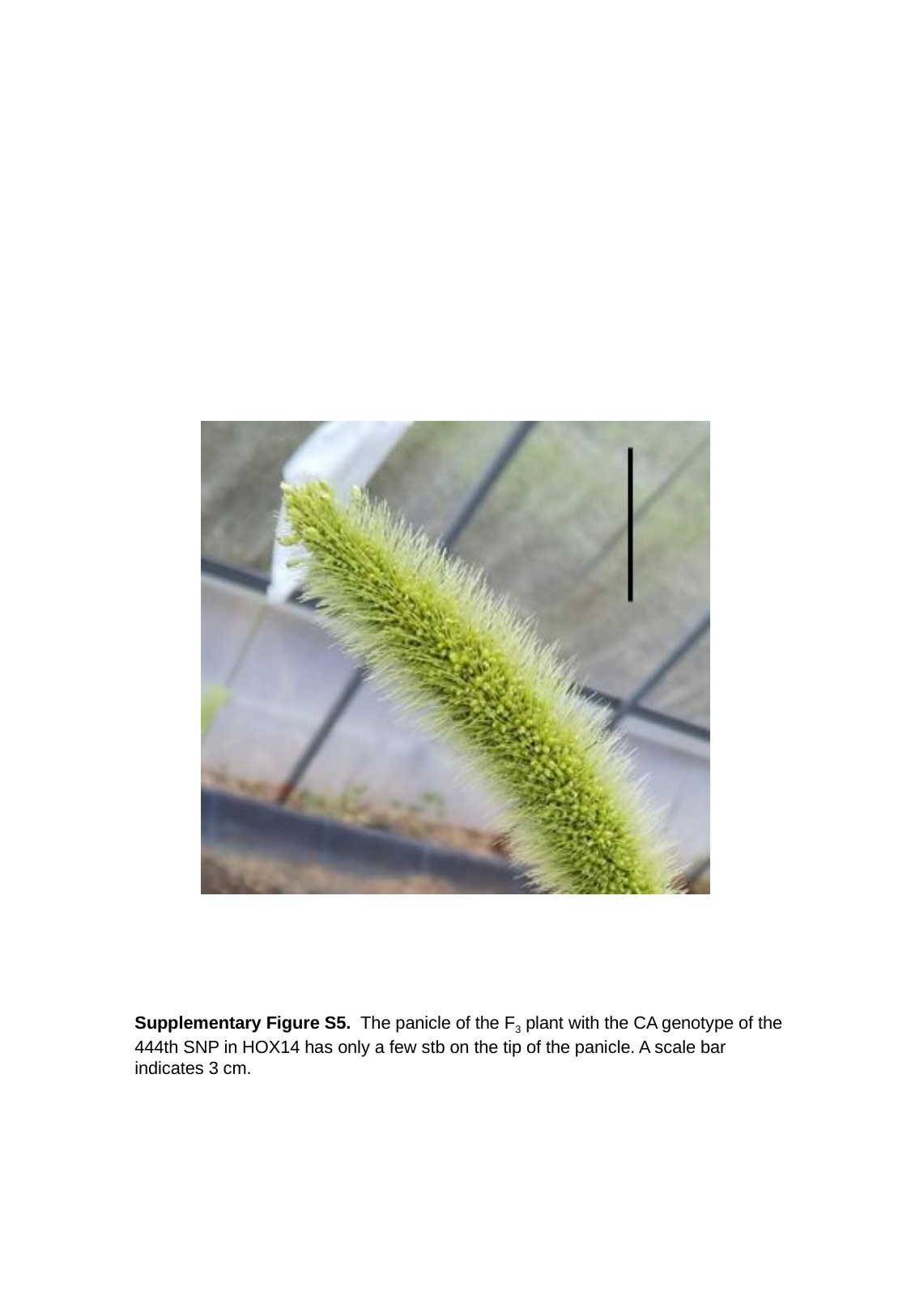

Supplementary Figure S5. The panicle of the F3 plant with the CA genotype of the 444th SNP in HOX14 has only a few stb on the tip of the panicle. A scale bar indicates 3 cm.

## Slide 6
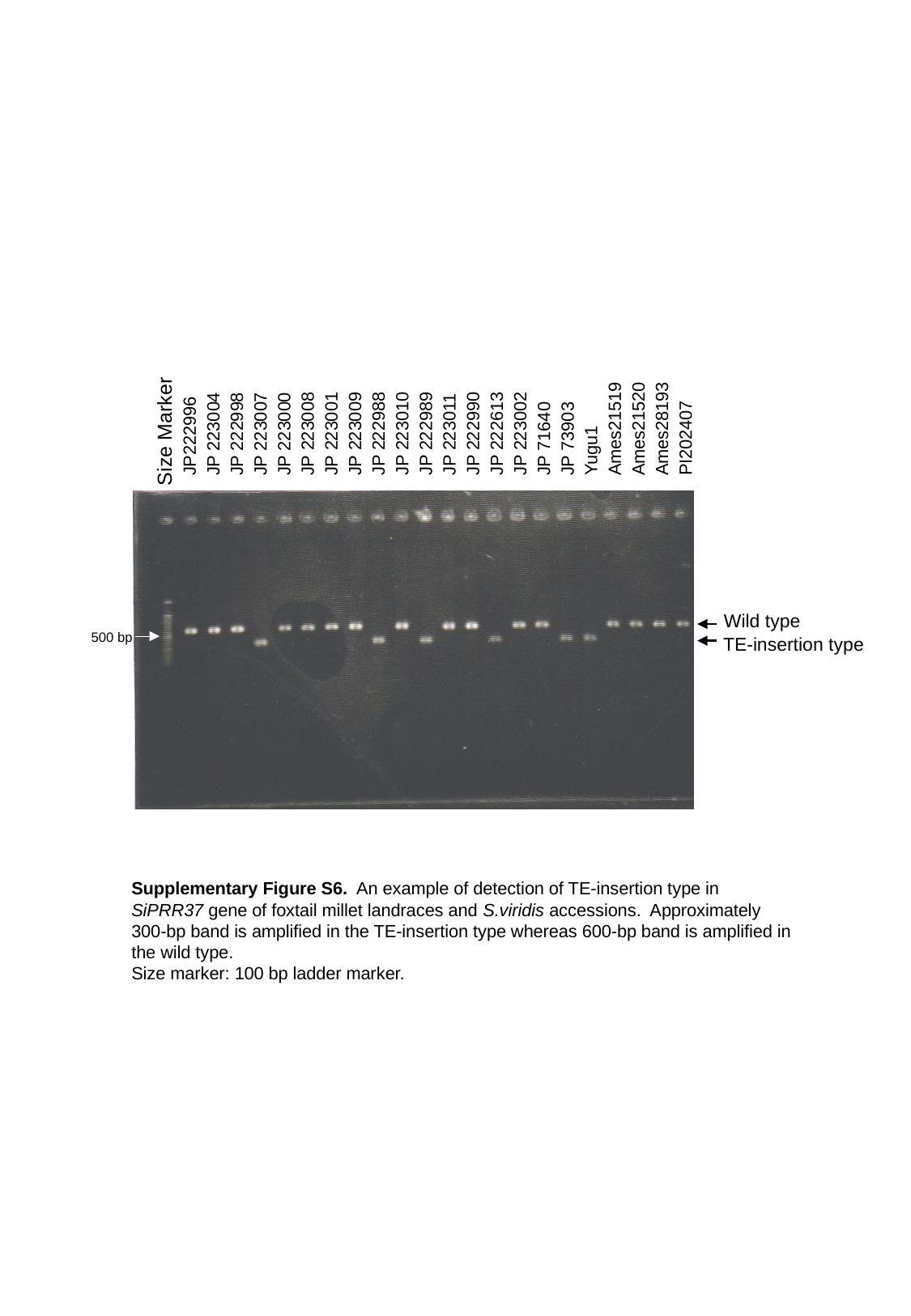

JP222996
JP 223004
JP 222998
JP 223007
JP 223000
JP 223008
JP 223001
JP 223009
JP 222988
JP 223010
JP 222989
JP 223011
JP 222990
JP 222613
JP 223002
JP 71640
JP 73903
Yugu1
Ames21519
Ames21520
Ames28193
PI202407
Size Marker
Wild type
500 bp -
TE-insertion type
Supplementary Figure S6. An example of detection of TE-insertion type in SiPRR37 gene of foxtail millet landraces and S.viridis accessions. Approximately 300-bp band is amplified in the TE-insertion type whereas 600-bp band is amplified in the wild type.
Size marker: 100 bp ladder marker.

## Slide 7
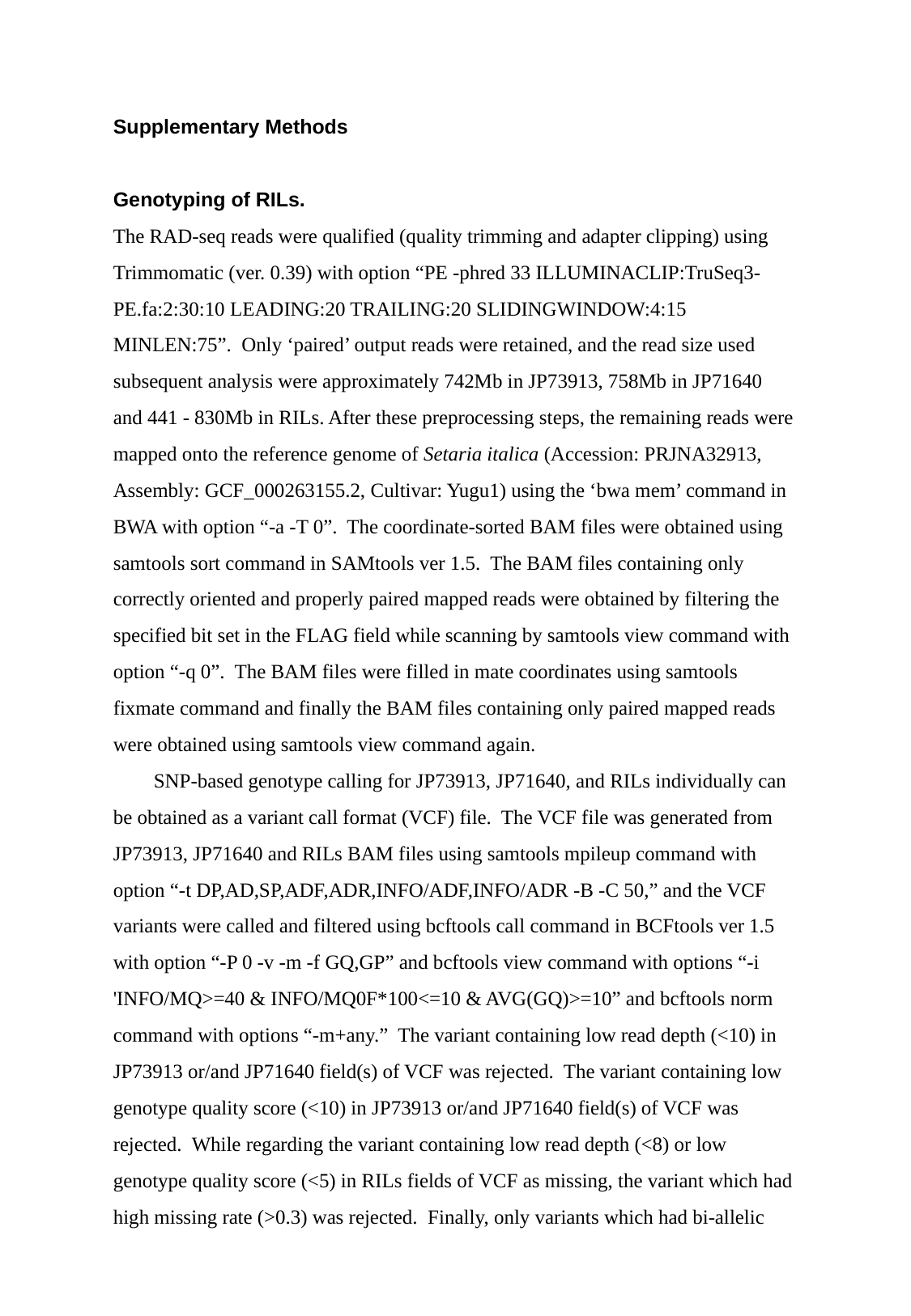

Supplementary Methods
Genotyping of RILs.
The RAD-seq reads were qualified (quality trimming and adapter clipping) using Trimmomatic (ver. 0.39) with option “PE -phred 33 ILLUMINACLIP:TruSeq3-PE.fa:2:30:10 LEADING:20 TRAILING:20 SLIDINGWINDOW:4:15 MINLEN:75”. Only ‘paired’ output reads were retained, and the read size used subsequent analysis were approximately 742Mb in JP73913, 758Mb in JP71640 and 441 - 830Mb in RILs. After these preprocessing steps, the remaining reads were mapped onto the reference genome of Setaria italica (Accession: PRJNA32913, Assembly: GCF_000263155.2, Cultivar: Yugu1) using the ‘bwa mem’ command in BWA with option “-a -T 0”. The coordinate-sorted BAM files were obtained using samtools sort command in SAMtools ver 1.5. The BAM files containing only correctly oriented and properly paired mapped reads were obtained by filtering the specified bit set in the FLAG field while scanning by samtools view command with option “-q 0”. The BAM files were filled in mate coordinates using samtools fixmate command and finally the BAM files containing only paired mapped reads were obtained using samtools view command again.
 SNP-based genotype calling for JP73913, JP71640, and RILs individually can be obtained as a variant call format (VCF) file. The VCF file was generated from JP73913, JP71640 and RILs BAM files using samtools mpileup command with option “-t DP,AD,SP,ADF,ADR,INFO/ADF,INFO/ADR -B -C 50,” and the VCF variants were called and filtered using bcftools call command in BCFtools ver 1.5 with option “-P 0 -v -m -f GQ,GP” and bcftools view command with options “-i 'INFO/MQ>=40 & INFO/MQ0F*100<=10 & AVG(GQ)>=10” and bcftools norm command with options “-m+any.” The variant containing low read depth (<10) in JP73913 or/and JP71640 field(s) of VCF was rejected. The variant containing low genotype quality score (<10) in JP73913 or/and JP71640 field(s) of VCF was rejected. While regarding the variant containing low read depth (<8) or low genotype quality score (<5) in RILs fields of VCF as missing, the variant which had high missing rate (>0.3) was rejected. Finally, only variants which had bi-allelic

## Slide 8
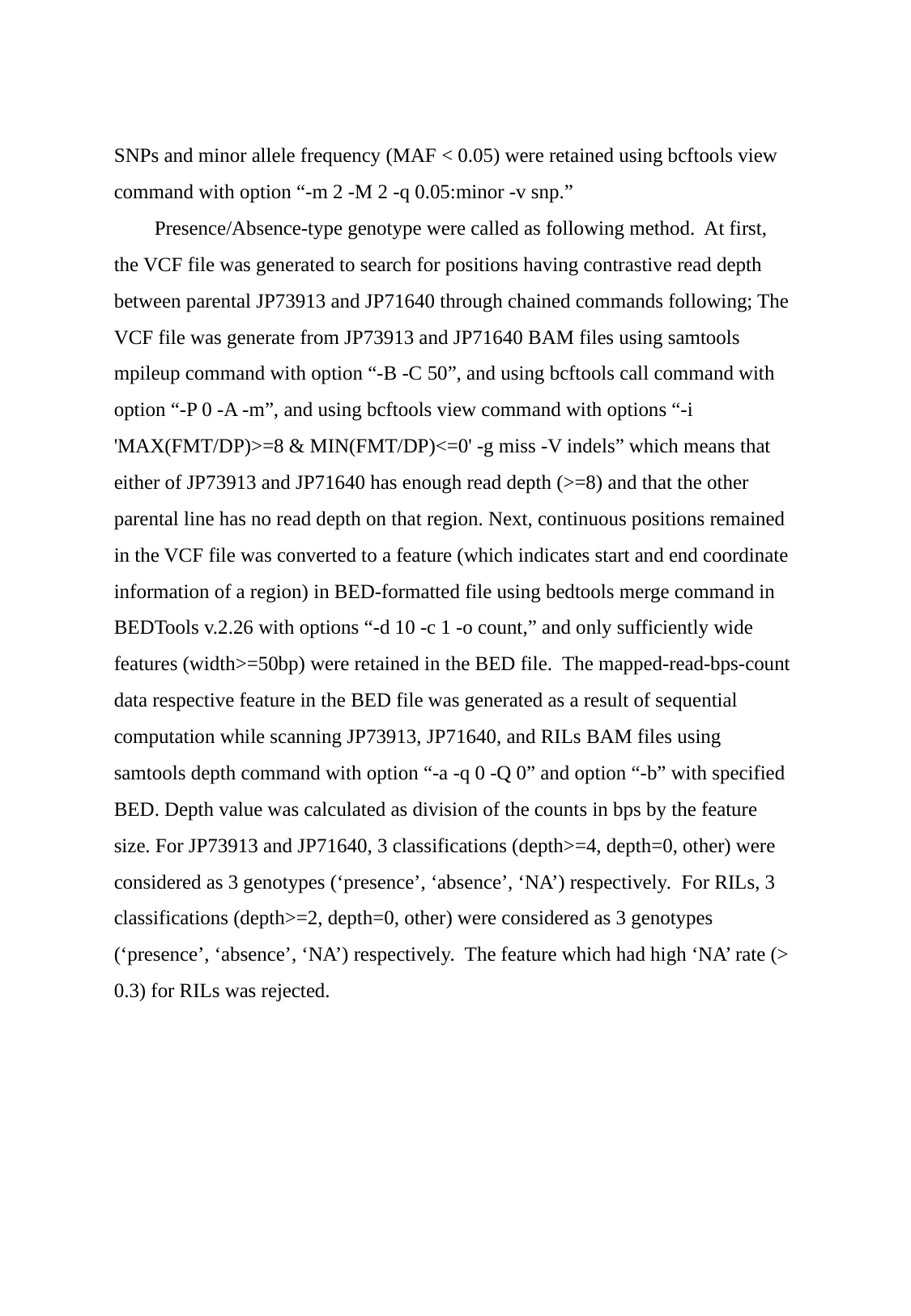

SNPs and minor allele frequency (MAF < 0.05) were retained using bcftools view command with option “-m 2 -M 2 -q 0.05:minor -v snp.”
 Presence/Absence-type genotype were called as following method. At first, the VCF file was generated to search for positions having contrastive read depth between parental JP73913 and JP71640 through chained commands following; The VCF file was generate from JP73913 and JP71640 BAM files using samtools mpileup command with option “-B -C 50”, and using bcftools call command with option “-P 0 -A -m”, and using bcftools view command with options “-i 'MAX(FMT/DP)>=8 & MIN(FMT/DP)<=0' -g miss -V indels” which means that either of JP73913 and JP71640 has enough read depth (>=8) and that the other parental line has no read depth on that region. Next, continuous positions remained in the VCF file was converted to a feature (which indicates start and end coordinate information of a region) in BED-formatted file using bedtools merge command in BEDTools v.2.26 with options “-d 10 -c 1 -o count,” and only sufficiently wide features (width>=50bp) were retained in the BED file. The mapped-read-bps-count data respective feature in the BED file was generated as a result of sequential computation while scanning JP73913, JP71640, and RILs BAM files using samtools depth command with option “-a -q 0 -Q 0” and option “-b” with specified BED. Depth value was calculated as division of the counts in bps by the feature size. For JP73913 and JP71640, 3 classifications (depth>=4, depth=0, other) were considered as 3 genotypes (‘presence’, ‘absence’, ‘NA’) respectively. For RILs, 3 classifications (depth>=2, depth=0, other) were considered as 3 genotypes (‘presence’, ‘absence’, ‘NA’) respectively. The feature which had high ‘NA’ rate (> 0.3) for RILs was rejected.
